# Editorial: Transforming Our World Through Events: The Agenda 2030

**DOI:** 10.3389/fspor.2022.942376

**Published:** 2022-06-24

**Authors:** Gayle McPherson, Laura Misener

**Affiliations:** ^1^School of Business and Creative Industries, Centre for Culture, Sport and Events, University of the West of Scotland, Paisley, United Kingdom; ^2^School of Kinesiology, Western University, London, ON, Canada

**Keywords:** mega-events, sport, Olympics, diplomacy, human-rights, soft power, legacy, UN SDG

Sporting and cultural events allow us the space to explore the contested terrain that events can act as a force for good and agents of change whilst acknowledging that the promises are often overstated and the evidence of change more limited (Misener et al., 2018). By engaging innovative ways of exploring the public value of events in relation to the United Nations Sustainable Development Goals and Agenda 2030, we witness the use of events as tools in positive social impacts, public and sport diplomacy, advocating for human rights, addressing issues of climate change, managing sustainability, and emphasizing social justice (Collison et al., 2019). The Sustainable Development Goals address global challenges including poverty, inequality, climate change, environmental degradation, peace and justice (Giulianotti, 2021). This agenda presents fruitful conceptual ground in aspects of public policy and global governance of sport mega and major events. Events can offer an avenue to address the global challenges by calling on governments and non-governmental organizations to account, empowering communities and citizens to have voice in the processes of change and ensure the potential soft power of sport and cultural diplomacy leads to sustainable change.

There is growing recognition that events and festivals take place in public and civic spaces, adding to public life, and that they have the potential to leave a lasting positive social legacy. This affords us the opportunity to engage in debates and dialogue that explore how events add to public value, how they can show normative consensus, how they can be used as progressive opportunities and how they can benefit global citizens who at times are marginalized and excluded. Mega and major events theoretically offer the potential to foster just and inclusive societies which are free from fear and violence. Yet, as we have seen in most recent times around mega sport events, the challenge to make good on these promises of peace is becoming even more challenging. Sport mega-events offer the vehicle for aspirations aligning with the SDGs albeit not without controversy.

This Research Topic has produced four thought-provoking intertwined manuscripts that offer different perspectives on the debates about the role of sport and events in supporting the SDGs. Each contribution offers unique viewpoint that can help inform policy makers, governments and event agencies of the power and disempowerment of events in contributing to global challenges that lie ahead. The approaches to understanding the role of sport and events are shaped around legacy expectations and planned externalities perceived from host citizens from mega-sport events, the hybrid threat covid-19 played to global society evidenced through the response to the Tokyo Olympics, the shortcomings of hosting mega sports events with the potential for associated abuses of human rights and lastly, the potential of virtual reality to be positioned as a new avenue for understanding social legacies of disability to broader impacts of events.

The four manuscripts provided in this first collection of the Sport, Leisure and Tourism section of the journal offer an interesting mix of critical realism and future potential of the role of events in addressing the goals of the UN SDG's and national/federal government's agenda for legacy and sustainability. Oshimi et al. present and extend the understanding of the role of social impact as a predictor variable for residents' behavior and intention to support events by using panel data before and after the Rugby World Cup in Japan. Their study extends the knowledge on consumer expectancy roles which are critical for host cities seeking to support the use of events for broader social agendas. Their study contributes to extending the use of social exchange theory in sporting events by identifying which impacts are more effective in forming residents' positive intention toward the event. Prospective host cities can look to this work to understand how to position the positive social impact potential of hosting an event.

Byers et al. break new ground in innovative approaches to considering social impact and the ways in which new technologies can support positive social legacies. They offer the possibility of the using virtual reality as tool for understanding disability leading to more opportunities for social inclusion. They specifically examine the potential of virtual reality as a technological innovation which can help create a social inclusion legacy in the context of Paris 2024 Olympic and Paralympic Games. Their conceptual model identifies legacy as a “wicked problem,” one in which host cities struggle with to develop outcomes that are realizable. They argue that we need to be embracing innovation with regards to legacy, by suggesting a new application for virtual reality technology in the context of legacy related to social inclusion. Investment in different approaches to create social legacies could help future host cities realize their desires to positive social impacts.

Ilevbare and McPherson present a conceptual and somewhat controversial discussion of the term hybrid threat and offers an re-conceptualization of the term to include COVID-19. Their discussion highlights how COVID-19 as a hybrid threat, affected Japan's ability to use the Tokyo 2020 Olympic Games as a soft power tool. The global pandemic realities threatened the very foundations of what the Olympics stood for, as a force for good. Further, the authors suggest that this hybrid threat in the Games environment was symbolic of the broader global vulnerabilities calling into question the basis for Governmental planning of the use of sports events in soft power strategies. The resulting effect is that Japan was unable to re-establish the soft power narratives that are typically brought about through the hosting of the Games. Thus, the Games failed to be the act of public diplomacy that it was intended to be when Tokyo acquired the events.

Finally, Ross and McDougall suggest a broader need to situate sport within the field of International Relations by outlining civil and human rights intrusions across successive Games. Their works suggest that the IOC, their partners and host cities are wedded in a symbolic and symbiotic courtship that manufactures local consent for and normalizes human right infringements. This all occurs while simultaneously providing the architecture for the spread and imposition of neoliberal order on the citizenry, masking the damage done by and through the hosting of the Olympics and Paralympic Games. The authors attest that the Olympics can leave a shadow legacy of damage for host cities, in particular what they describe as vulnerable cities. But the paper is not doomsday cry for the Games, rather it's a call for new theoretical understandings that bring together cultural sociology and entrepreneurship as an avenue for hope for the future of the Olympics.

This section of the journal was launched in early 2020, and this Research Topic along with the launch. Thus, getting submissions early on proved a challenge. Nonetheless, the breadth of manuscript herein is demonstrative of the need to explore and challenge the evidence of legacy, social impact and achievement toward the UN SDG's beyond the rhetoric of bid documents and political statements. Scholars are coming up with creative theoretical approaches and are offering innovative social impact opportunities that support the idea that events can be a force of transformation for good. But this work is not without its challenges and much more needs to be done. As we move into a post-COVID era of rebuilding sport and events, and reconciling the losses and opportunities of the past several years, the time is ripe with opportunity to critically assess where and how events will be part of the ongoing global transformation.

## Author Contributions

Both authors listed have made a substantial, direct, and intellectual contribution to the work and approved it for publication.

## Conflict of Interest

The authors declare that the research was conducted in the absence of any commercial or financial relationships that could be construed as a potential conflict of interest.

## Publisher's Note

All claims expressed in this article are solely those of the authors and do not necessarily represent those of their affiliated organizations, or those of the publisher, the editors and the reviewers. Any product that may be evaluated in this article, or claim that may be made by its manufacturer, is not guaranteed or endorsed by the publisher.

## References

[B1] Collison H. Darnell S. Giulianotti R. Howe P. D. (2019). The Routledge Handbook of Sport for Development and Peace. London: Routledge.

[B2] Giulianotti R.. (2021). Greening sport for development and peace: a socio-ecological approach. *Front. Sports Act*. Living. 3, 122. 10.3389/fspor.2021.660743 34124657 PMC8193491

[B3] Misener L. McPherson G. McGillivray D. Legg D. (2018). Leveraging Disability Sport Events: Impacts, Promises, and Possibilities. Oxon: Routledge.

